# Non-K Region Disubstituted Pyrenes (1,3-, 1,6- and 1,8-) by (Hetero)Aryl Groups—Review

**DOI:** 10.3390/molecules24142551

**Published:** 2019-07-12

**Authors:** Dawid Zych

**Affiliations:** 1 Institute of Chemistry, Faculty of Mathematics, Physics and Chemistry, University of Silesia, Szkolna 9, 40-007 Katowice, Poland; dawidzych92@gmail.com; 2 Institut für Silizium-Photovoltaik, Helmholtz-Zentrum Berlin für Materialien und Energie GmbH, Kekuléstraße 5, 12489 Berlin, Germany

**Keywords:** pyrene chemistry, synthetic methods, substitution pattern, bromination, acylation, coupling reactions, condensation, cycloaddition

## Abstract

Disubstituted pyrenes at the non-K region by the same or different (hetero)aryl groups have proven to be an increasingly interesting area of research for scientists over the last decade due to their optical and photophysical properties. However, in this area, there is no systematization of the structures and synthesis methods nor their limitations. In this review, all approaches to the synthesis of these compounds, starting from the commercially available pyrene are described. Herein, the ways of obtaining of disubstituted intermediates based on bromination and acylation reaction are presented. This is crucial in the determination of the possibility of further functionalization by using coupling, cycloaddition, condensation, etc. reactions. Moreover, the application of disubstituted pyrenes in the synthesis of 1,3,6,8-tetrasubstituted was also reviewed. This review describes the directions of research on chemistry of disubstituted pyrenes.

## 1. Introduction

Despite the topic of pyrene derivatives having already been covered extensively by scientific literature, it still proves to be a popular subject of new research [1,2,3]. Without a doubt, pyrene and its derivatives exhibit intriguing properties. Multiple systematic studies have shown this already, yet still, new areas of interest such as the non-K region (the positions 4-, 5-, 9-, and 10- of pyrene are described as K-region due to carcinogenic effect of pyrene upon its oxidation) disubstituted by aryl or heteroaryl groups at pyrenes (1,3-, 1,6-, and 1,8-) are being elucidated. Disubstituted pyrenes of this type are interesting in themselves and can act as substrates in the synthesis of the other molecules that also exhibit expected properties. The vast majority of disubstituted pyrenes can be applied in organic electronics in materials such as organic light-emitting diodes (OLEDs) [4,5,6,7,8,9], organic field-effect transistors (OFETs) [10,11], and solar cells [12] but also in the synthesis of nanographenes [13], metal cages [14,15], and many others. A wide spectrum of methods for the synthesis of the reported compounds exists, though a fundamental problem lies within the methods’ ordering. Nonetheless, in 2011, Klaus Müllen and Teresa M. Figueira-Duarte presented a review article about pyrene-based materials for organic electronics [1], in 2014, Anthony P. Davis et al. systematized the ways of synthesis of substituted pyrenes by indirect methods [16], and in 2016, Xing Feng et al. described functionalization of pyrene in detail, especially tetrasubstituted pyrenes at non-K and K-region, which are suitable as luminescent materials [2]. However, the systematization of 1,3-, 1,6-, and 1,8-disubstituted pyrenes is still lacking.

**Figure 1:**
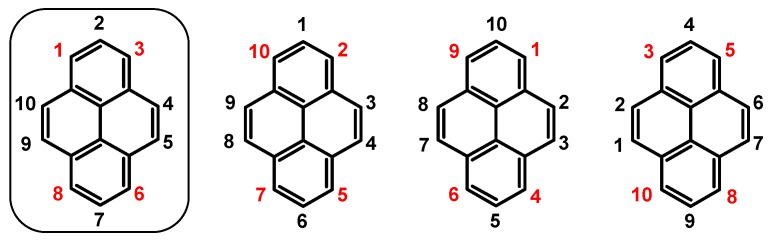
Various locant numeration for pyrene structure.

Despite the hard work of the scientists mentioned above, an issue concerning the description of substituted positions in recently published papers on pyrenes becomes apparent. Indeed, it could just be a result of getting used to an idea replicated in literature. However, if a recognized misconception is accepted as truth, it ought to be eliminated and corrected. According to International Union of Pure and Applied Chemistry (IUPAC) enumeration, what was also mentioned by Franz S. Ehrenhauser, [17] it should be as presented in Figure 1 for pyrene in the frame.

In the presented review, the ways of synthesis of 1,3-, 1,6- and 1,8-disubstituted pyrenes starting from pyrene, followed by the intermediates such as dibromo, diacetyl, and boroorganic pyrenes suitable for further functionalization in pure form or as mixtures are described as reported in the literature. Moreover, the possibility of the application of disubstituted pyrenes in the synthesis of 1,3,6,8-tetrasubstituted is also presented.

## 2. Dibromopyrenes

The most significant role in the synthesis of disubstituted pyrenes plays its dibromo derivatives, which are suitable for the further functionalization in various reactions such as substitution and coupling reactions (Suzuki-Miyaura, Stille, and Sonogashira). The electronic structure of pyrene causes a bromination reaction, and the derivatives containing bromine at positions 1-, 3-, 6-, 8- (non-K region) can be preferably obtained. Only the application of appropriate reaction conditions allows us to obtain dibromopyrenes with the expected substitution pattern.

### 2.1. 1,6- And 1,8-dibromopyrene

**Scheme 1:**
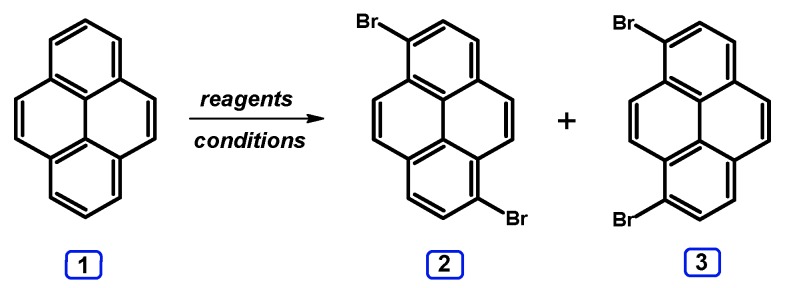
Introduction of bromine into the pyrene structure at positions 1,6- and 1,8-.

The interest of the synthesis and obtaining of the 1,6- and 1,8-dibromopyrene (Scheme 1) in its pure form dates back to early 1970s of the previous century when J. Grimshaw and J. Trocha-Grimshaw reported a procedure for synthesis that used slow addition of bromine solution in carbon tetrachloride into pyrene **1** solution in the same solvent, which resulted in the isomers that were separated by crystallization from toluene or mixture of benzene-hexane with 44% yield 1,6-isomer **2** and 45% yield 1,8-isomer **3** [18].

In the following years, various solvents, brominating agents, and reaction conditions were used. The vast majority of reported procedures was focused on obtaining the pure 1,6-isomer (Table 1). It can be noted that, in the case of carbon disulfide used as a solvent, the 1,8-isomer is obtained with the high yield ~85%. What is more, in other cases, almost the same reaction conditions resulted in the products with yields varying about 40%, which means the main problem is connected with the purification of the crude mixture after the reaction’s completion.

**Scheme 2:**
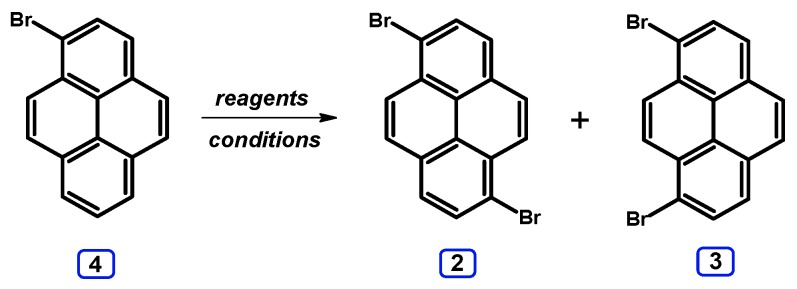
Bromination of 1-bromopyrene.

The other approach to the synthesis of 1,6- and 1,8-dibromopyrene presented in the literature is based on the synthesis in which the starting material 1-bromopyrene **4** is used (Scheme 2). 1-Bromopyrene can be successfully obtained with the high yield up to 96% by bromination of pyrene by the mixture HBr/H_2_O_2_ [34].

The reaction conditions for the method mentioned above of obtaining of 1,6- and 1,8-dibromopyrene are discussed in the literature in two publications. The first used a mixture of KBr/NaClO in HCl and MeOH solution, yielding in a mixture of products with 43% yield, whereas in the second case, bromine in dichloromethane obtained the target pure dibromopyrenes, with yields about 35% for every isomer (Table 2).

### 2.2. 1,3-Dibromopyrene

As presented above, the synthesis of 1,6- and 1,8-dibromopyrenes is well described, whereas the 1,3-isomer is relatively unexplored. It is related to the difficulty of substitution of the pyrene structure due to the preference for electrophilic substitution at the 1,6- and 1,8- positions rather than the 1,3-positions of pyrene. The determined spectroscopy yield of that isomer that is present as a byproduct of the bromination reaction equals 3% [36]. It causes that the substitution at positions 1 and 3 is only possible by the multistep reactions with the number of intermediates that contain the protecting groups at 7-position. 2-Pyrenecarboxylic acid **5** is suitable for that reaction and can be obtained in two multistep ways—starting from 4,5,9,10-tetrahydropyrene [37] or pyrene [38].

**Scheme 3:**
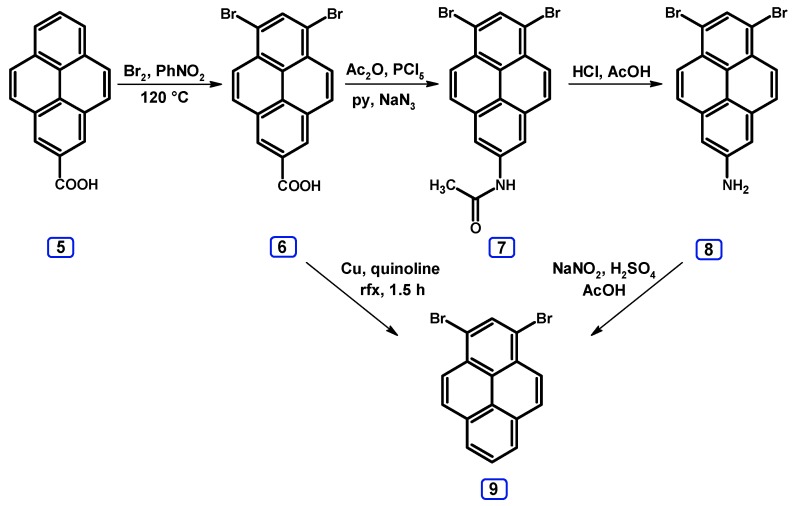
Synthetic routes of obtaining 1,3-dibromopyrene.

In the first approach reported in 1972 by Yu. E. Gerasimenko, 2-pyrenecarboxylic acid **5** was used in the bromination reaction, obtaining 1,3-dibromo-7-pyrenecarboxylic acid **6**, which in further steps turned into 1,3-dibromo-7-aminopyrene **8**, followed by the Sandmeyer reaction, which resulted in 1,3-dibromopyrene **9** with a 9.3% yield (Scheme 3) [39]. The other synthesis possibility was described by T. Nielsen et al., where 1,3-dibromopyrene was prepared from 1,3-dibromo-7-pyrenecarboxylic acid **6**, previously obtained in alkaline hydrolysis of methyl 1,3-dibromopyrene-2-carboxylate. Intermediate **6** is used in the decarboxylation reaction with copper powder in boiling quinoline [40]. It should be noticed that authors describing the usage of 230 g of **6**, resulting in 120 mg of **9**. It can be supposed that 10 mL of quinoline and 100 mg of copper powder would be suitable for 230 mg of **6**. I also conducted the reaction on the scale of 230 mg of **6** and 100 mg of Cu powder, but the target product was not obtained. Nontrivial synthesis of 1,3-dibromopyrene and the insufficiently reported protocols of its synthesis are also demonstrable by the lack of its application in the synthesis of 1,3-disubstituted pyrenes; only the approach with acylation of pyrene allows us to obtain the 1,3-disubstituted pyrenes, as described above.

### 2.3. Suzuki-Miyaura Coupling

**Scheme 4:**
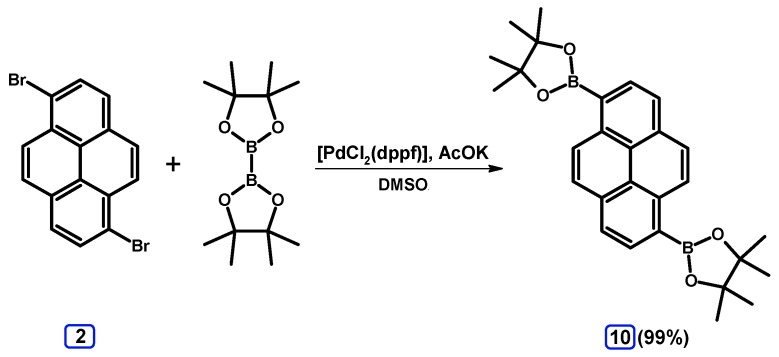
Synthesis of 1,6-bis(4,4,5,5-tetramethyl-1,3,2-dioxaborolane-2-yl)pyrene [41].

**Scheme 5:**
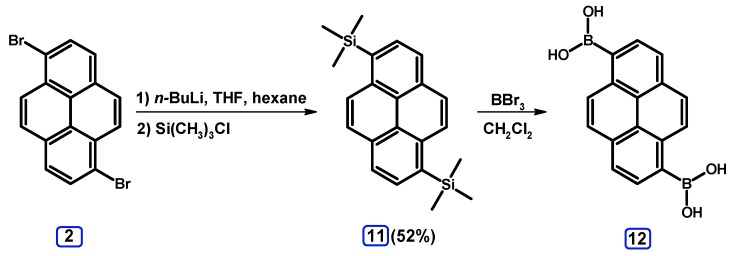
Synthesis of 1,6-pyrenediyldiboronic acid [30].

Dibromopyrenes (1,6- and 1,8-isomer) are most often used in Suzuki-Miyaura coupling reaction in which they can react with (hetero)arylboronates or (hetero)arylboronic acids as well as after the functionalization as a boroorganic compounds. The synthesis of boroorganic derivatives of pyrene was described for 1,6-isomer (Scheme 4 and Scheme 5). 1,6-Bis(4,4,5,5-tetramethyl-1,3,2-dioxaborolane-2-yl)pyrene **10** can be obtained in the commonly used reaction between the bromo derivative with bis(pinacolato)diboron in the presence of the catalyst [PdCl_2_(dppf)] and AcOK as a base, which results in a product with 99% yield [41].

Hikaru Suenaga et al. described the synthesis of 1,6-pyrenediyldiboronic acid that was obtained in a two-step reaction starting from 1,6-dibromopyrene and followed by 1,6-bis(trimethylsilyl)pyrene intermediate **11**, which was suitable for obtaining the target acid **12** (Scheme 5). The authors did not report the yield of the compound **12** because it was used directly in the synthesis of pyrene-1,6-diyldiboronic acid dimethyl ester, which was obtained with a 61% yield [30].

#### 2.3.1. Pyrene Derivative Acting as a Boroorganic Compound

**Scheme 6:**
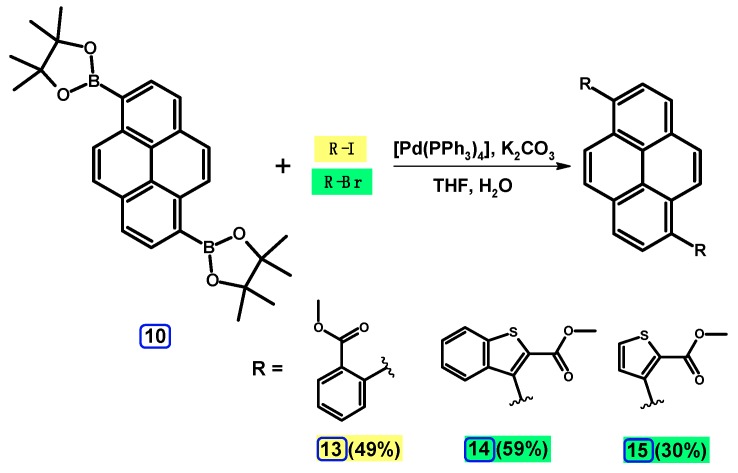
The Suzuki-Miyaura coupling reactions of **10** [10,42].

The application of the boroorganic derivative of pyrene **10** was presented by Long Chen and co-workers. Molecule **10** was applied in the reaction with methyl 2-iodobenzoate with the catalytic system [Pd(PPh_3_)_4_]/K_2_CO_3_ in THF/H_2_O, which resulted in the derivative **13** with 49% yield (Scheme 6) [10]. This compound was used further in the synthesis of the angularly fused bistetracene. Compound **10** was also reacted with bromo derivatives of methyl benzo[b]thiophene-2-carboxylate or methyl thiophene-2-carboxylate that yielded **14** (59%) and **15** (30%), which were used in the synthesis of bisthienoacenes [42].

**Scheme 7:**
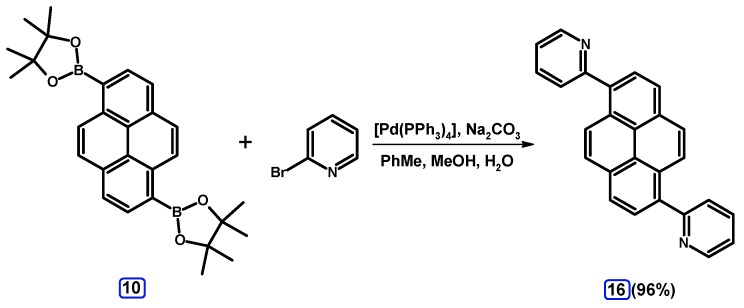
Suzuki-Miyaura coupling reaction to afford **16** [41].

Xinliang Feng et al. reported the synthesis of 1,6-di(pyridin-2-yl)pyrene based on the Suzuki-Miyaura coupling reactions between **10** and 2-bromopyridine with catalytic system [Pd(PPh_3_)_4_]/Na_2_CO_3_ in PhMe/MeOH/H_2_O, which obtained product **16** with 96% yield (Scheme 7) and which was further used in synthesis of target cationic nitrogen-doped helical nanographenes [41].

**Scheme 8:**
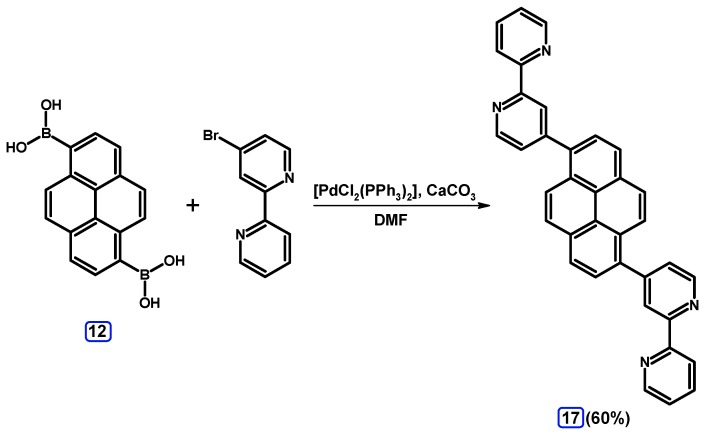
Suzuki-Miyaura reaction with 1,6-pyrenediyldiboronic acid **12** [43].

There is only one report where 1,6-pyrenediyldiboronic acid is used in the synthesis of the pyrene derivative containing substituted heteroaryl groups. In that case, 1,6-bis(bipyridinyl)pyrene **17** was synthesized by using the system [PdCl_2_(PPh_3_)_2_]/CaCO_3_ in DMF with 4-bromo-2,2′-bipyridine, which resulted in a product with 60% yield (Scheme 8) [43].

#### 2.3.2. Suzuki-Miyaura Coupling of Dibromopyrenes with (Hetero)Arylboronic Acids

**Scheme 9:**
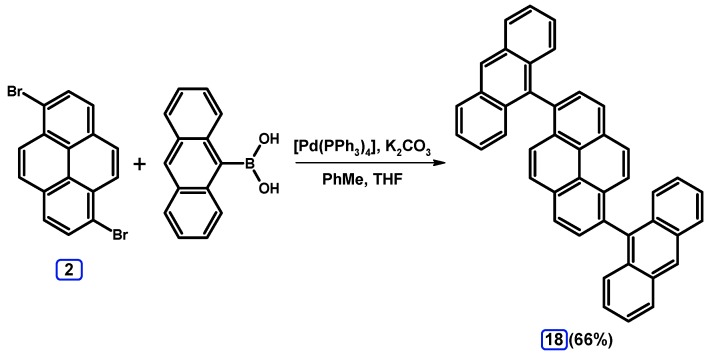
Introduction of anthracen-9-yl motifs into pyrene structure by using the Suzuki-Miyaura coupling reaction [7].

Plenty of the reports are dedicated to the Suzuki-Miyaura coupling reactions where dibromopyrenes react with (hetero)arylboronic acids. The introduction of anthracen-9-yl motifs into a pyrene structure was presented by Jongwook Park et al., where [Pd(PPh_3_)_4_]/K_2_CO_3_ in PhMe/THF was used as a catalytic system (Scheme 9). It resulted in 1,6-di(anthracen-9-yl)pyrene **18** with a 66% yield, which was used in the preparation of organic emitter films [7].

**Scheme 10:**
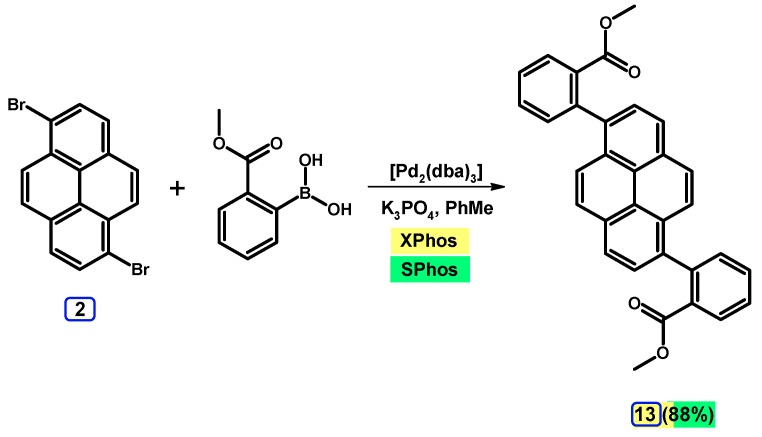
Synthesis route to compound **13** [44,45].

Due to the wide interest in organic semiconductors based on the expanded polyaromatic structures such as bistetracene and naphtho-tetracenone, molecule **13**, which is suitable for their synthesis, was also obtained by Michel Frigoli and co-workers using 2-methoxycarbonylphenylboronic acid with catalytic system [Pd_2_(dba)_3_]/K_3_PO_4_ in PhMe with two kinds of phosphines—SPhos and XPhos (Scheme 10). [44,45] The results of the reactions did not show any differences in the yield of the product (88%) in reference to using phosphine. It should be noted that the presented method resulted in a product with a higher yield of about 39% in comparison to the report of Long Chen et al. [10].

**Scheme 11:**
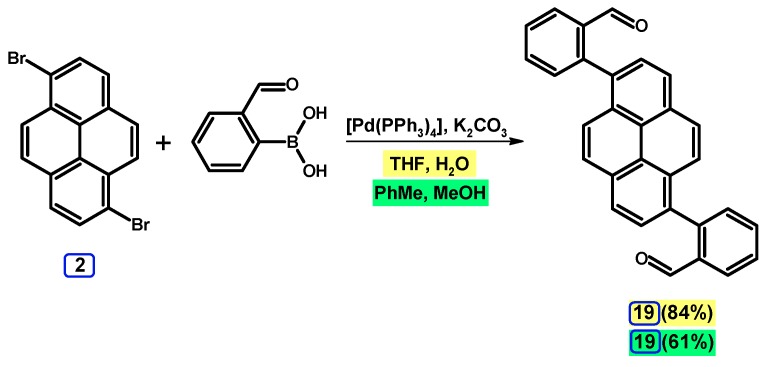
Synthesis of 1,6-bis(2-formylphenyl)pyrene **19** [13,46].

Among the other important disubstituted pyrene derivatives that are necessary for the synthesis of nanographenes, 1,6-bis(2-formylphenyl)pyrene **19** plays an important role. The compound mentioned above was obtained by two research teams (Scheme 11). Both of them used catalytic system [Pd(PPh_3_)_4_]/K_2_CO_3_ but different solvents. Wenming Su et al. carried out the reaction in a mixture of THF/H_2_O which led to obtaining a product with a higher yield (84%) [46] in comparison to Konstantin Amsharov et al., who applied a mixture of PhMe/MeOH, obtaining a product with 61% yield [13].

**Scheme 12:**
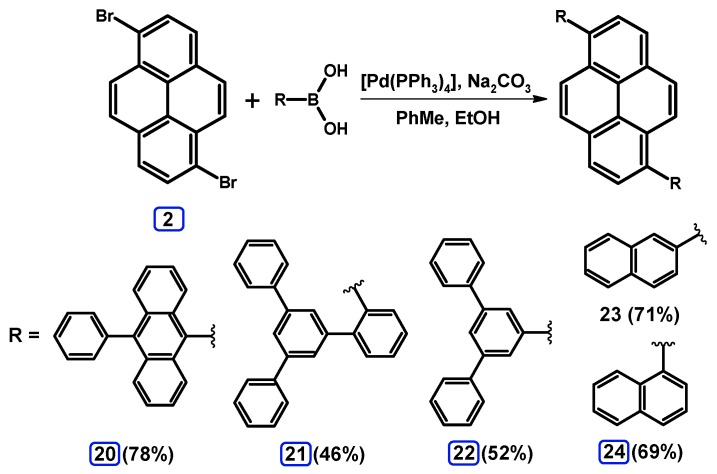
Suzuki-Miyaura coupling reaction resulted in molecules **20**–**24** [4,47,48].

**Scheme 13:**
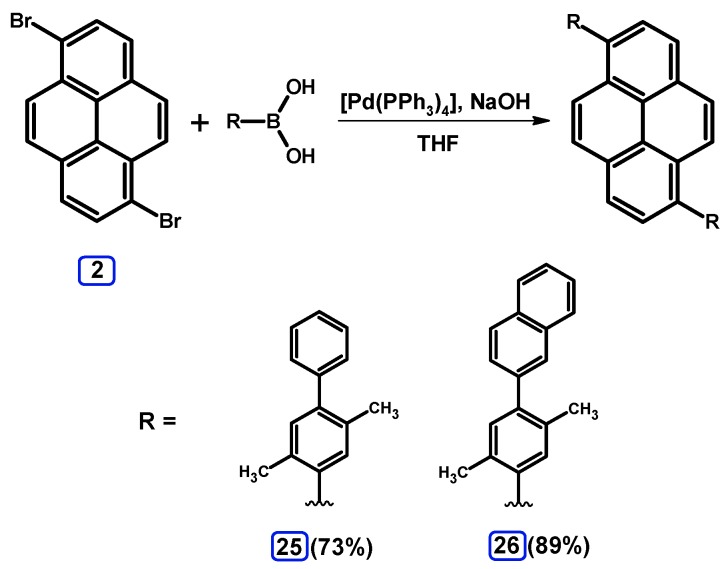
Suzuki-Miyaura coupling reaction resulted in molecules **25**, **26** [49].

**Scheme 14:**
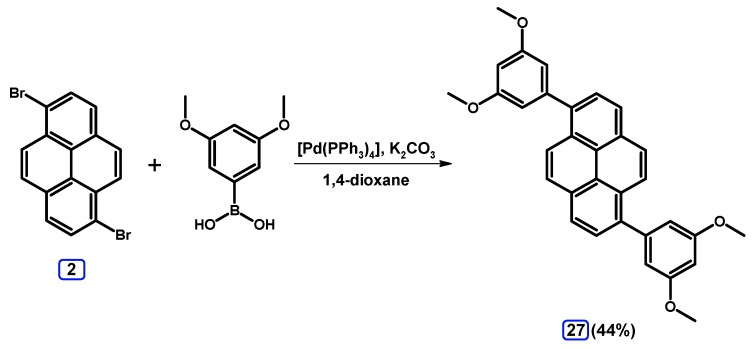
Suzuki-Miyaura coupling reaction resulted in molecule **27** [8].

**Scheme 15:**
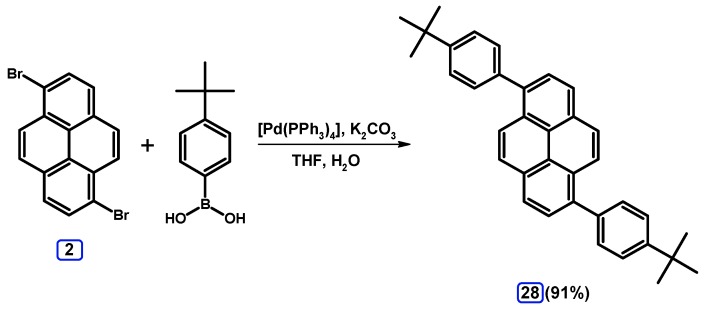
Suzuki-Miyaura coupling reaction resulted in molecule **28** [50].

Investigation of the efficient organic light-emitting devices based on pyrene derivatives was also a stimulus to the synthesis of 1,6-disubstituted pyrenes, which contain various aryl groups **20**–**28**, such as presented in Scheme 12, Scheme 13, Scheme 14 and Scheme 15 [4,8,47,48,49,50]. All reactions used [Pd(PPh_3_)_4_] as a catalyst and are divided in reference to applied bases Na_2_CO_3_, NaOH, and K_2_CO_3_, and solvents PhMe/EtOH, 1,4-dioxane, and THF.

#### 2.3.3. Suzuki-Miyaura Coupling of Dibromopyrenes with (Hetero)Arylboronates

**Scheme 16:**
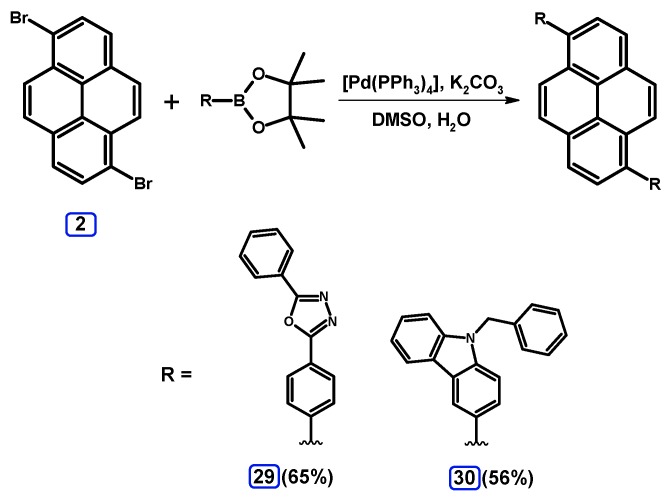
Obtaining compounds **29** and **30** in coupling reaction with (hetero)arylboronates [51].

The synthesis of the next part of reported disubstituted pyrene derivatives was also conducted using the Suzuki-Miyaura coupling reaction, but in this case, with (hetero)arylboronates. The aim of the synthesis was similar—obtaining the most efficient materials for OLEDs or molecules that will be used in further functionalization. Liheng Feng et al. presented two 1,6-disubstituted pyrenes containing 4-(5-phenyl-1,3,4-oxadiazol-2-yl)phenyl **29** and 9-benzyl-9*H*-carbazol-2-yl **30** groups and described by them as pyrenes substituted at positions 2 and 7 (Scheme 16) [51]. The catalytic system [Pd(PPh_3_)_4_]/K_2_CO_3_ in DMSO/H_2_O was used, which resulted in the products **29** and **30** with yields of 65% and 56%, respectively.

**Scheme 17:**
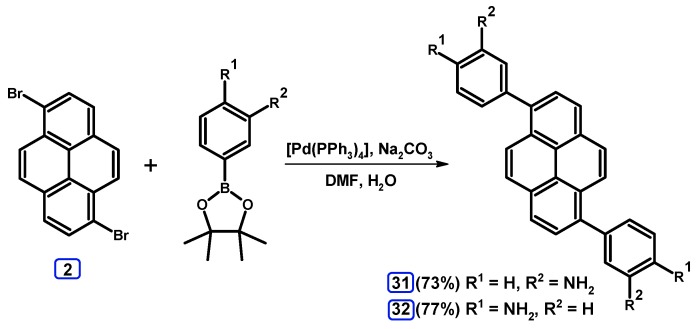
Synthesis of 1,6-bis(4-aminophenyl)pyrene **31** and 1,6-bis(3-aminophenyl)pyrene **32** [14,15].

In 2014 and 2015, Jonathan R. Nitschke and co-workers published two papers about the pyrene-edged cages where 1,6-bis(4-aminophenyl)pyrene **31** or 1,6-bis(3-aminophenyl)pyrene **32** were used as a starting material [14,15] As the authors described, molecules **31** and **32** were obtained in the presence of Pd(PPh_3_)_4_]/Na_2_CO_3_ in DMF/H_2_O with similar yields of about 75% (Scheme 17).

**Scheme 18:**
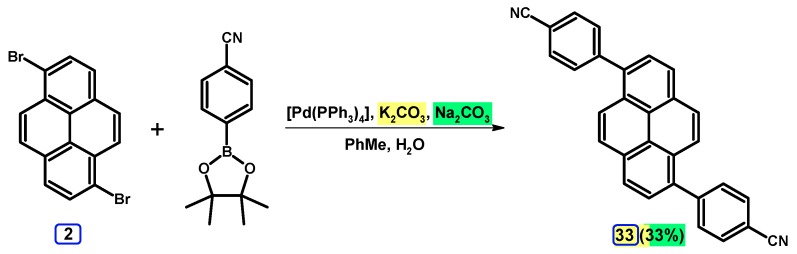
Obtaining of disubstituted pyrene containing 4-cyanophenyl unit **33** [20,52].

**Scheme 19:**
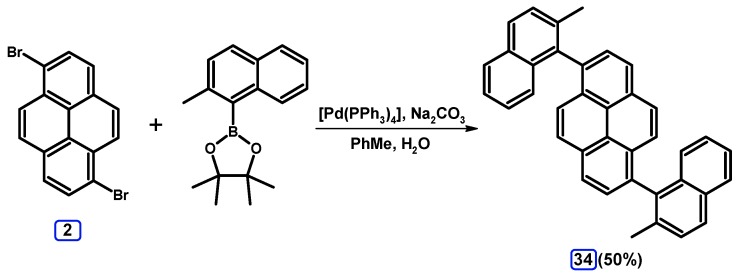
Obtaining of compound **34** [53].

**Scheme 20:**
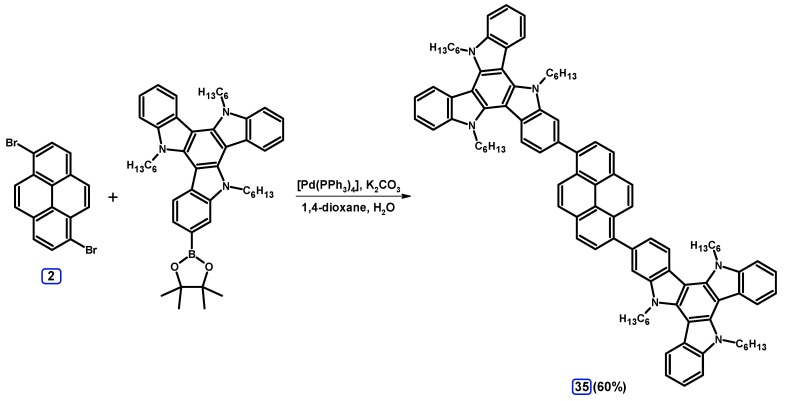
Synthesis of molecule **35** [54].

Among the described derivatives of 1,6-disubstituted pyrene, the group of molecules containing 4-cyanophenyl **33** [20,52], 2-methyl-1-naphthyl **34** [53], or expanded groups based on diindolocarbazole **35** [54] were obtained using [Pd(PPh_3_)_4_] with base K_2_CO_3_ or Na_2_CO_3_ in PhMe/H_2_O or 1,4-dioxane/H_2_O solution, which resulted in target products with yields up to 60% (Scheme 18, Scheme 19 and Scheme 20).

**Scheme 21:**
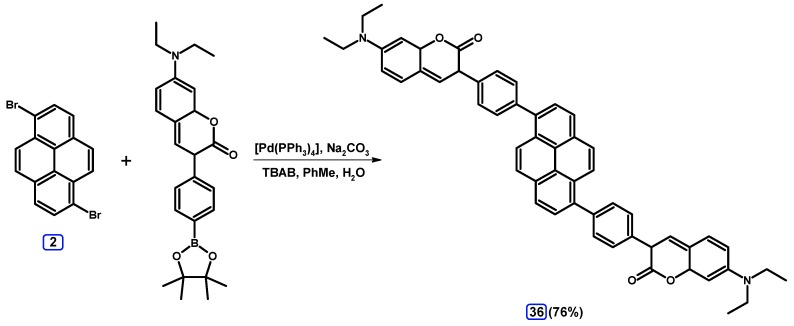
Synthesis of compound **36** [55].

In the case of synthesis of the compound containing phenylcoumarin **36**, a small excess of tetrabutylammonium bromide (TBAB) (5% mol) was used, which significantly increased the yield of the reaction, and the product was obtained with a 76% yield (Scheme 21) [55].

**Scheme 22:**
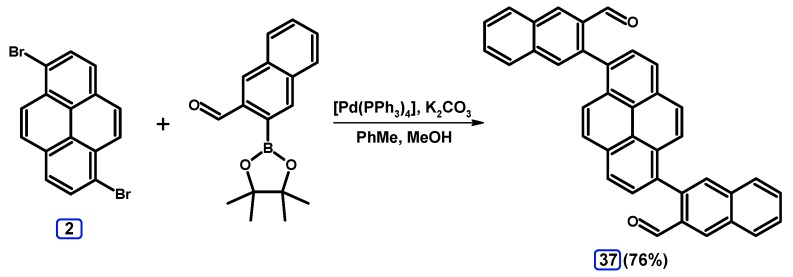
Synthesis of 1,6-bis(3-formylnaphthyl)pyrene **37** [13].

The previously mentioned research team of Konstantin Amsharov also reported the synthesis of 1,6-bis(3-formylnaphthyl)pyrene, which, in contrast to disubstituted pyrene by 2-formylphenyl groups, was obtained in the Suzuki-Miyaura coupling reaction with 3-formylnaphthalene-2-boronic acid pinacol ester, which resulted in **37** with a higher yield of 76% (Scheme 22) [13].

**Scheme 23:**
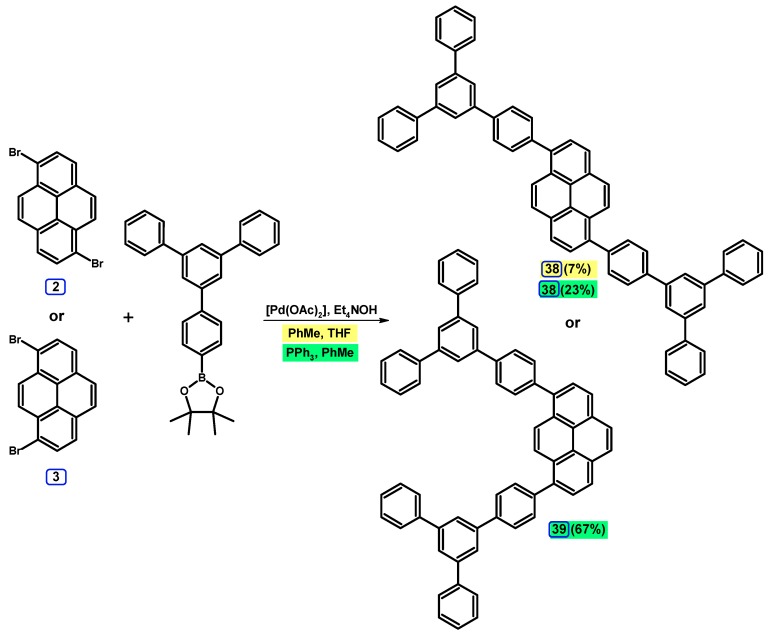
Synthesis of 1,6- and 1,8-disubstituted pyrenes **38** and **39** [24,25].

In 2016, Jongwook Park and co-workers obtained 1,6-bis(3,5-diphenylbiphenyl-4-yl)pyrene **38** by using the system [Pd(OAc)_2_]/Et_4_NOH in PhMe/THF what resulted in a product with low yield ≈ 7% (Scheme 23) [24]. Two years later, the same team presented extensive research with molecule **38** and its 1,8- and 4,9- isomers, which were synthesized starting from the pure dibromopyrene isomers using the catalytic system [Pd(OAc)_2_]/Et_4_NOH in PhMe with the addition of triphenylphosphine (PPh_3_). As a result of the reaction, molecule **38** was obtained with a 16% higher yield (23%), whereas the 1,8-isomer **39** had a 67% yield [25].

**Scheme 24:**
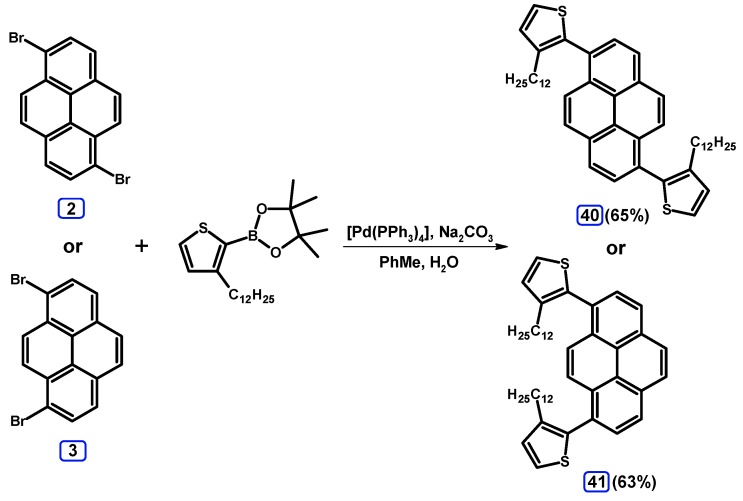
Obtaining of 1,6- and 1,8-isomers containing dodecylthiophen-2-yl substituents **40** and **41** [21].

Introduction of the 3-dodecylthiophen-2-yl units into pyrene at positions 1,6- and 1,8- was described by Deqing Gao et al., where, as a starting material, pure dibromopyrenes were applied and reacted with dodecylthiophene-2-boronic acid pinacol ester using [Pd(PPh_3_)_4_] with base Na_2_CO_3_ in PhMe/H_2_O solution, which resulted in products with comparable yields 65% and 63% for 1,6-bis(3-dodecylthiophen-2-yl)pyrene **40** and 1,8-bis(3-dodecylthiophen-2-yl)pyrene **41**, respectively (Scheme 24) [21].

**Scheme 25:**
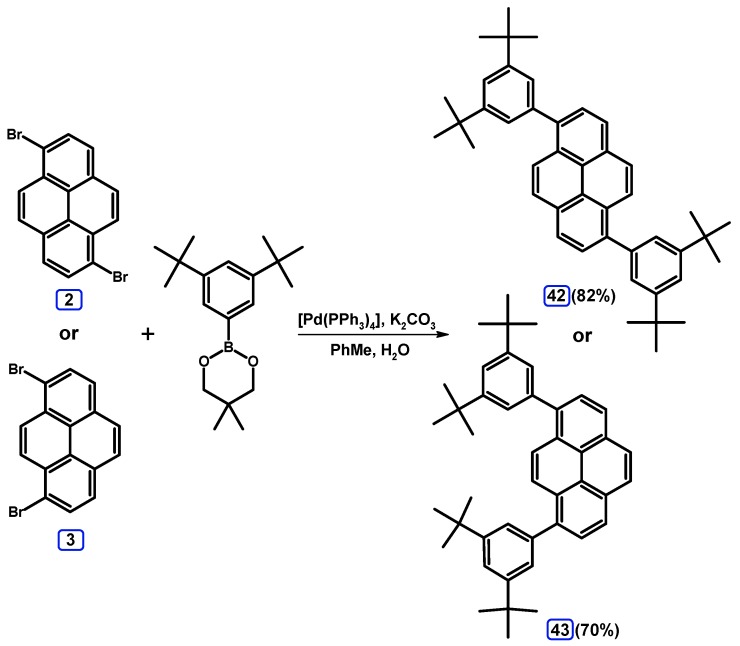
Suzuki-Miyaura coupling reaction with 5,5-dimethyl-2-(3,5-di-*tert*-butylphenyl)-1,3,2-dioxaborinane [56].

Based on similar reaction conditions, Yoshiteru Sakata introduced 3,5-di-*tert*-butylphenyl substituents into 1,6- and 1,8- positions of pyrene using 5,5-dimethyl-2-(3,5-di-*tert*-butylphenyl)-1,3,2-dioxaborinane, which resulted in molecules **42** and **43** with 82% and 70% yields, respectively (Scheme 25) [56].

#### 2.3.4. Mono-Suzuki-Miyaura Coupling

**Scheme 26:**
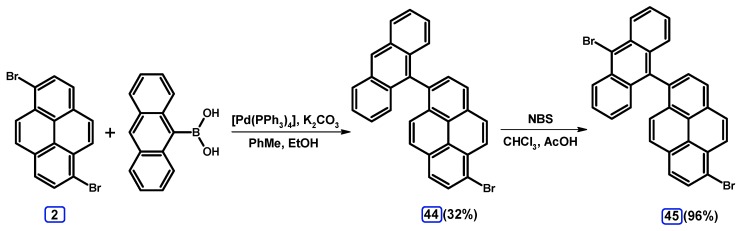
Introduction of anthracen-9-yl motif by using mono-Suzuki-Coupling reaction [22].

The pioneer in applying the mono-Suzuki-Miyaura coupling reactions in the synthesis of asymmetric 1,6-disubstituted pyrenes is Jongwook Park and co-workers, who presented in several papers derivatives of pyrene that contain at 1-position anthracen-9-yl motif (mostly substituted at 10-position) and at 6-position various aryl/heteroaryl groups. The introduction of anthracen-9-yl group into the pyrene structure was achieved by the Suzuki-Miyaura coupling of anthracene-9-boronic acid with 1,6-dibromopyrene, where the boroorganic compound was used with 1.5 excess, which resulted in **44** with a 32% yield (Scheme 26). The further functionalization of the obtained compound was possible by bromination reaction using *N*-bromosuccinimide (NBS), which resulted in **45** with a 96% yield.

**Scheme 27:**
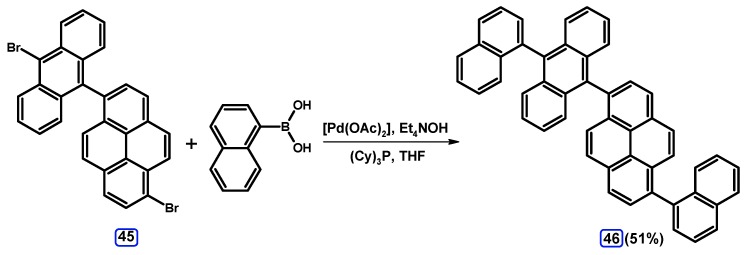
Synthesis of 1,6-disubstituted unsymmetrical pyrene derivative **46** [22,57].

**Scheme 28:**
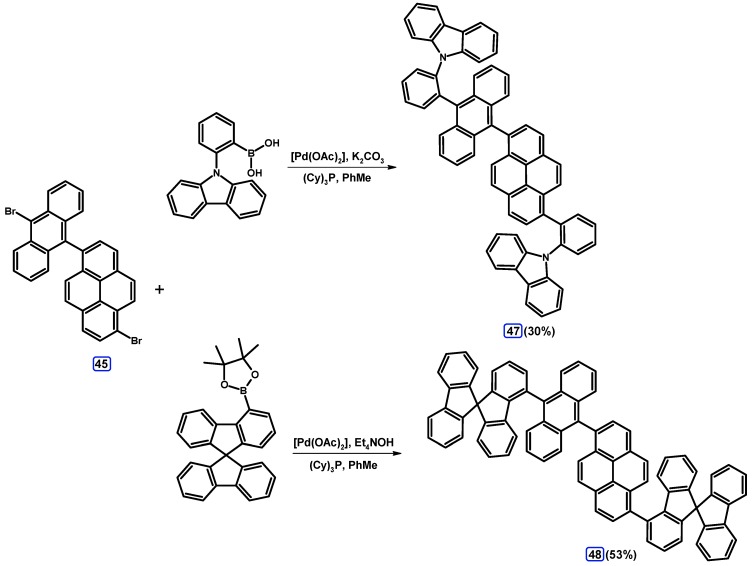
Obtaining of the 1,6-disubstituted pyrene derivatives with one substituted anthracenyl unit **47** and **48** [58].

**Scheme 29:**
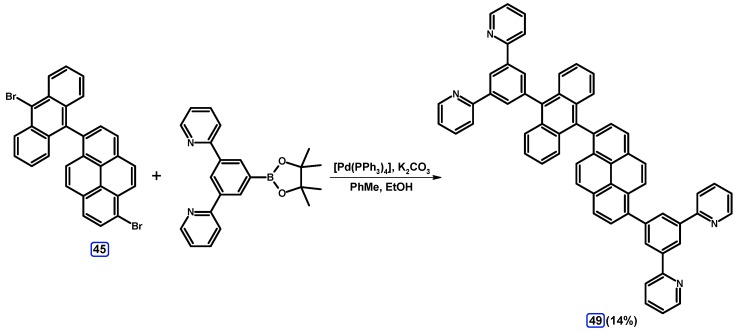
Synthesis of molecule **49** [59].

Obtained intermediate **45** was used in the next reactions of the introduction of aryls at the 6-position of pyrene and also at 10-position of the substituted anthracen-9-yl group, which was conducted using Suzuki-Miyaura coupling with various boroorganic derivatives, i.e., boronic acids or boronates (Scheme 27, Scheme 28 and Scheme 29). The catalytic systems based on [Pd(OAc)_2_]/Et_4_NOH resulted in molecules **46** and **48** with higher yields—i.e., 51% and 53%, respectively—in comparison to system [Pd(OAc)_2_]/K_2_CO_3_ and [Pd(PPh_3_)_4_]/K_2_CO_3_ for molecules **47** (30%) and **49** (14%) [22,57,58,59].

**Scheme 30:**
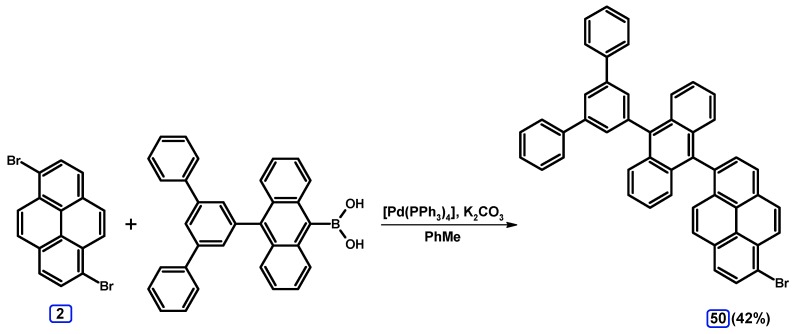
Suzuki-coupling reaction with an expanded anthracen-9-yl substituent [60].

**Scheme 31:**
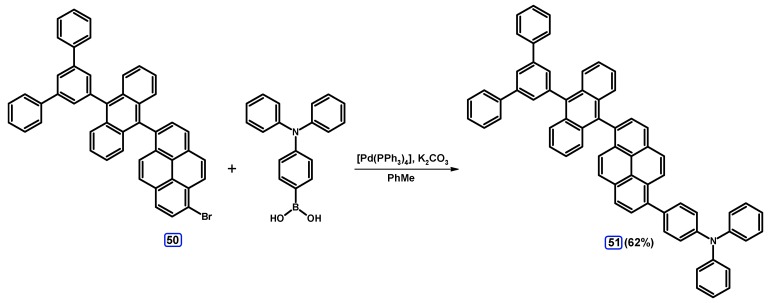
Introduction of triphenylamine by using Suzuki-coupling reaction [60].

Furthermore, Jongwook Park et al. reported mono Suzuki-Miyaura coupling with already substituted anthracen-9-yl at 10-position by 1,1’:3’,1’’-terphenyl-5’-yl unit, which resulted in **50** with a 42% yield (Scheme 30) [60]. Further functionalization of molecule **50** was achieved by the introduction of triphenylamine substituent, which resulted in **51** with a 62% yield (Scheme 31).

**Scheme 32:**
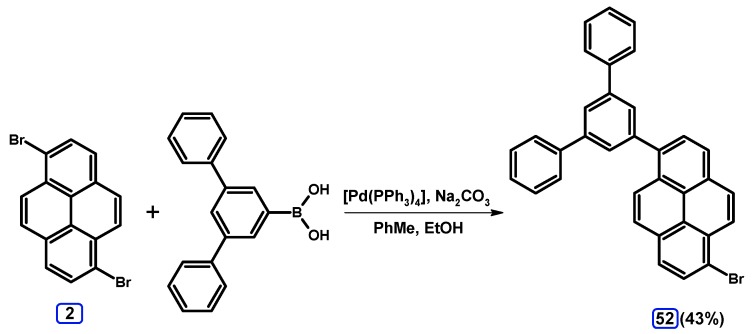
Mono Suzuki-Miyaura coupling reaction resulted in compound **52** [61].

**Scheme 33:**
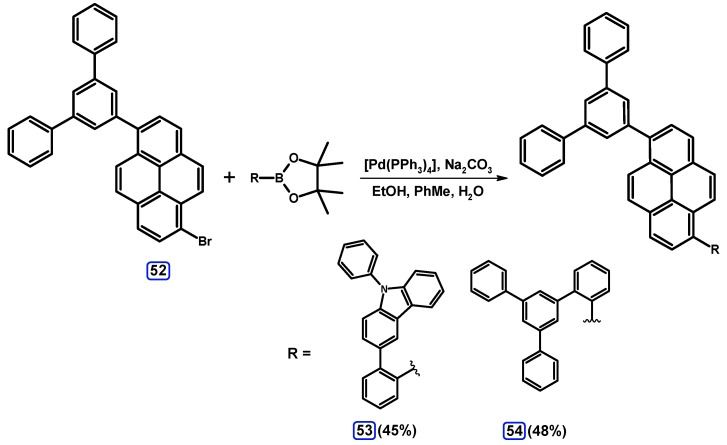
Further functionalization of **52** by the Suzuki-Miyaura coupling reaction [61].

In the same year, Baoming Ji and co-workers published a paper with unsymmetrical 1,6-disubstituted pyrene derivatives where the starting monosubstituted pyrene was substituted by 1,1’:3’,1’’-terphenyl-5’-yl unit **52**, which was obtained with 43% yield (Scheme 32) [61]. Molecule **52** was subjected in the next reaction with boronic acids pinacol ester, which allowed them to introduce 3-(2-phenyl)-9-phenylcarbazole **53** and 5′-phen-2-yl-1,1′:3′,1″-terphenyl **54** at 6-position groups with 45% and 48% yields, respectively (Scheme 33).

### 2.4. Stille Coupling

**Scheme 34:**
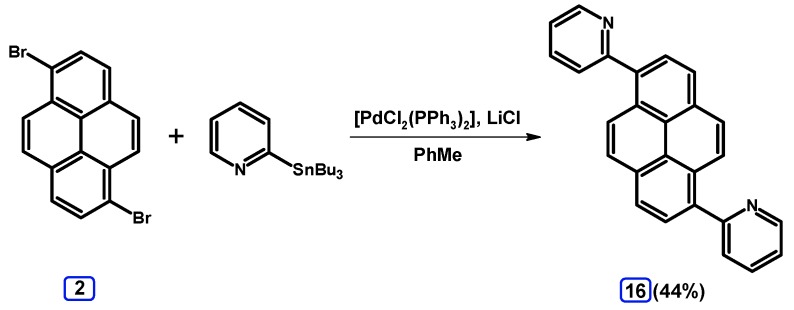
Stille cross-coupling reaction in obtaining of 1,6-di(pyrid-2-yl)pyrene **16** [62].

In the area of disubstituted pyrene derivatives obtained using the Stille coupling reaction, there are only three papers in which authors used tributylstannyl derivatives of heteroaryls. The other approach to synthesis of previously mentioned 1,6-di(pyrid-2-yl)pyrene **16** was reported by Yu-Wu Zhong and Yan-Qin He in the presence of [PdCl_2_(PPh_3_)_2_], LiCl in PhMe, which resulted in a product with a significantly lower yield of 44% (Scheme 34). Synthesis using the Suzuki-Miyaura reaction obtained a product with a 96% yield (Scheme 7) [62].

**Scheme 35:**
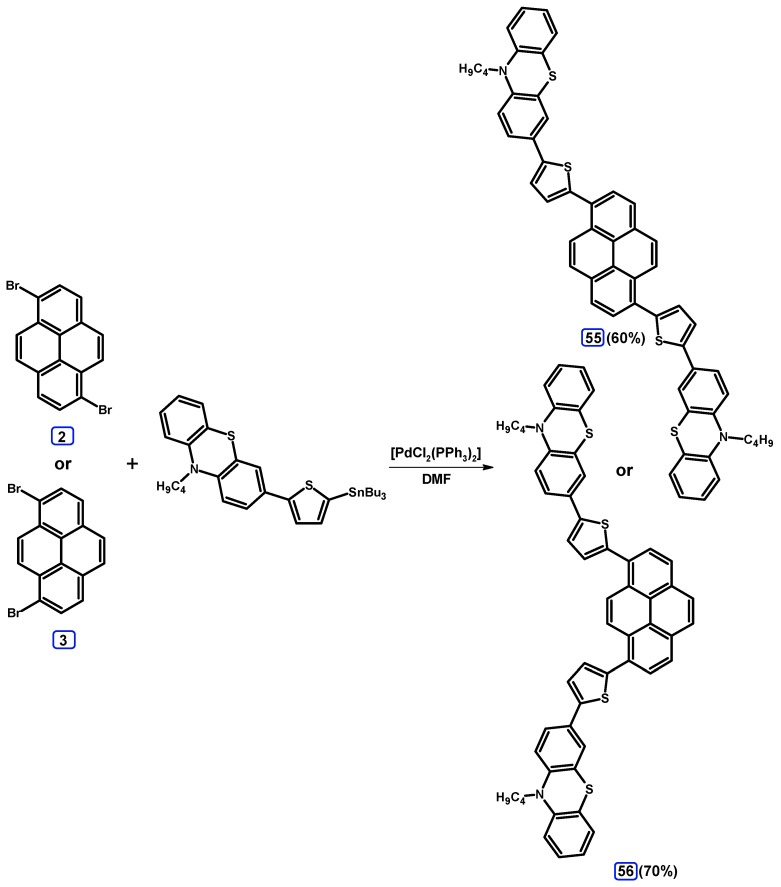
Introduction of thienylphenothiazine by using the Stille-coupling reaction [63].

K. R. Justin Thomas and co-workers reported two isomers of pyrene derivative (1,6- and 1,8-) that are substituted by thienylphenothiazine groups and that were obtained starting from pure dibromo isomers of pyrene using [PdCl_2_(PPh_3_)_2_] as a catalyst in DMF solution, which resulted in products **55** and **56** with high yields of 60% and 70%, respectively (Scheme 35) [63].

**Scheme 36:**
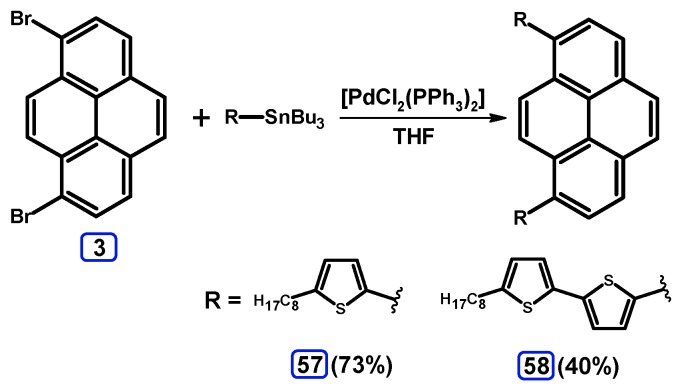
Synthesis of 1,8-bis(5-octyl-2-thienyl)pyrene **57** and 1,8-bis[5 -octyl-(2,2′-bithiophen)-5′-yl]pyrene **58** [11].

1,8-Disubstituted pyrenes dedicated for materials that can be used as high-performance organic field-effect transistors containing 5-octyl-2-thienyl **57** and 5-octyl-(2,2′-bithiophen)-5′-yl **58** substituents were obtained with 73% and 40% yields, as described by Deqing Gao et al. (Scheme 36) [11].

### 2.5. Sonogashira Coupling

**Scheme 37:**
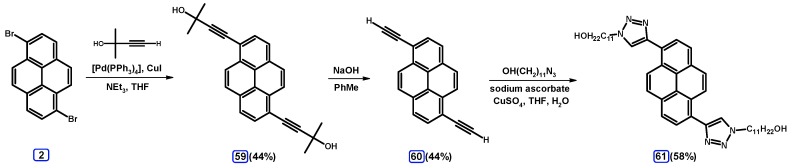
Sonogashira-coupling reaction followed by the Huisgen cycloaddition [64].

Applying the Sonogashira coupling reaction in the synthesis of disubstituted pyrenes containing directly substituted (hetero)aryl groups was described by Bo Song and co-workers [64]. The authors presented the synthetic route leading to 1,6-diethynylpyrene **60**, which was obtained in a two-step reaction with a 44% yield. That compound was suitable for the Huisgen cycloaddition reaction, which allowed for the synthesizing of pyrene substituted by triazolyl groups **61** (Scheme 37). It should be mentioned that, in the literature, other examples of disubstituted pyrenes by triazolyl groups are present, but the synthetic methodology is similar [65,66].

### 2.6. Ullmann, Buchwald-Hartwig, Rosenmund-von Braun, and Substitution Reactions

**Scheme 38:**
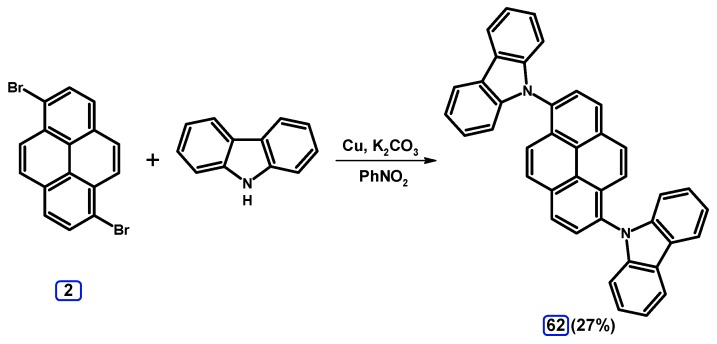
Ullmann reaction [67].

Another important approach to the synthesis of disubstituted pyrenes is based on Ullmann C-N coupling reaction, described by Yoon Soo Han and co-workers, where 1,6-di(9*H*-carbazol-9-yl)pyrene **62** was obtained at the presence of Cu/K_2_CO_3_ in PhNO_2_, which resulted in a product with 27% yield (Scheme 38) [67].

**Scheme 39:**
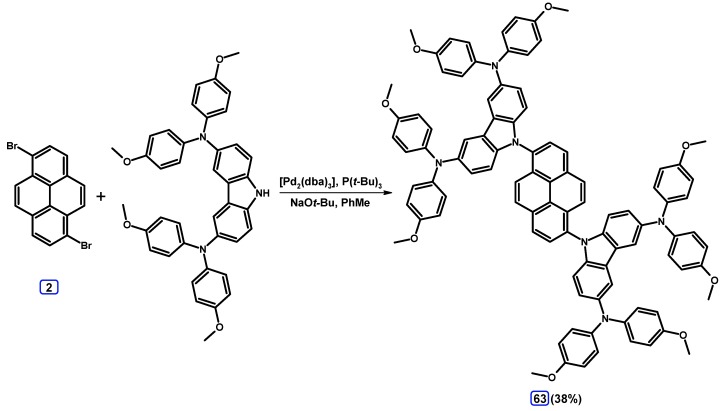
Buchwald-Hartwig cross-coupling reaction [12].

Synthesis of disubstituted pyrenes in which substituents are connected by the C-N bond can also be obtained by the Buchwald-Hartwig cross coupling reaction, which was reported by Qingbo Meng et al. for 1,6-disubstituted by N3,N6-bis(di-4-anisylamino)-9*H*-carbazole groups **63**. This was obtained by using the catalytic system [Pd_2_(dba)_3_]/P(*t*-Bu)_3_/NaO*t*-Bu in PhMe with a 38% yield (Scheme 39) [12].

**Scheme 40:**
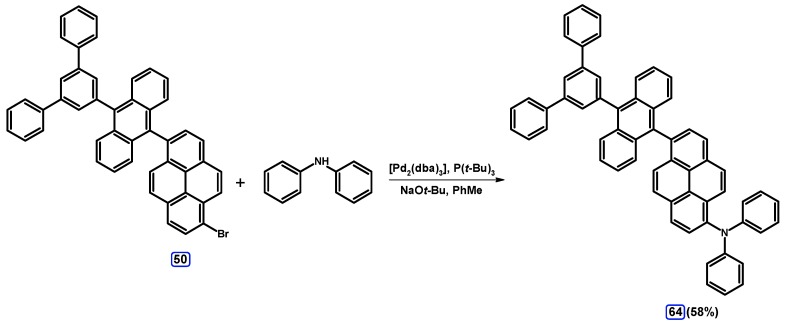
Further functionalization of **51** by Buchwald-Hartwig cross-coupling reaction [60].

The same coupling reaction was also applied for the previously described product of mono Suzuki-Miyaura coupling reaction **50**, which allowed the introduction of the diphenylamine moiety into the structure at 6-position with a 58% yield **64** (Scheme 40) [60].

**Scheme 41:**
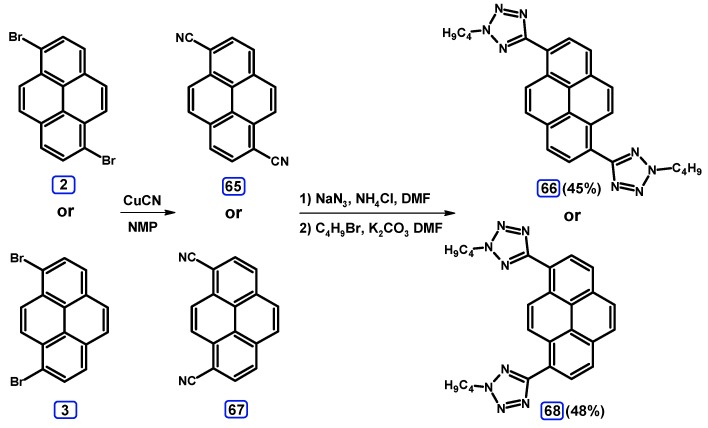
Rosenmund-von Braun reaction followed by cycloaddition reaction [68].

1,6- And 1,8-disubstituted pyrenes by 2-butyl-*2H*-1,2,3,4-tetrazol-5-yl groups **66** and **68** were synthesized starting from pure dibromo isomers in which, as the result of the Rosenmund-von Braun reaction using CuCN in NMP, bromine atoms were exchanged on cyano groups **65** and **67**. The obtained intermediates were suitable for the cycloaddition reaction [3 + 2] using NaN_3_/NH_4_Cl in a DMF solution, followed by the alkylation with butyl bromide. This resulted in molecules **66** and **68** with 45% and 48% yields (Scheme 41) [68].

**Scheme 42:**
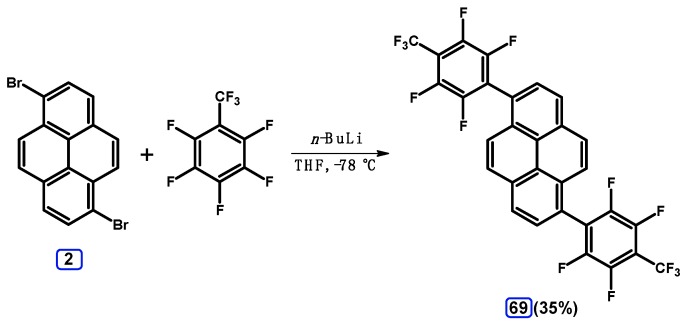
Synthesis of 1,6-di[2,3,5,6-tetrafluoro-4-(trifluoromethyl)phenyl]pyrene **69** based on the S_N_Ar process [52].

Deqing Gao and co-workers reported a synthesis route based on the nucleophilic aromatic substitution S_N_Ar, which used the lithiation of 1,6-dibromopyrene using *n*-BuLi in THF at −78 °C, which formed the carbanion. The obtained intermediate reacted with the large excess of octafluorotoluene, which resulted in 1,6-di[2,3,5,6-tetrafluoro-4-(trifluoromethyl)phenyl]pyrene **69** with a 35% yield (Scheme 42) [52].

### 2.7. Reaction with a Mixture of 1,6- and 1,8-dibromo Isomers

**Scheme 43:**
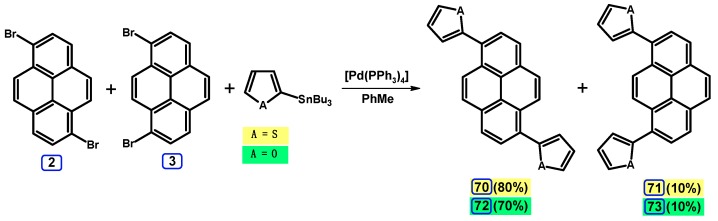
Stille-coupling reaction with a mixture of 1,6- and 1,8-dibromopyrene [69,70].

Depending on target molecules and separation possibility of isomers, there is also another approach to the synthesis of 1,6- and 1,8-disubstituted pyrenes, which was presented by three research teams. Krzysztof Idzik and co-workers described the spectrum of pyrene derivatives containing furyl and thienyl units substituted at various positions of pyrene. In the case of 1,6- and 1,8- isomers, as a starting material, the mixture of 1,6- and 1,8-dibromopyrenes (authors described the isomers as 1,6- and 1,4-) was applied in the Stille-coupling reaction with 2-(tributylstannyl)thiophene or 2-(tributylstannyl)furan. This resulted in mixtures of isomers that were isolated using column chromatography, yielding compounds **70** (80%) and **71** (10%) in the case of thienyl units and **72** (70%) and **73** (10%) containing furyl groups (Scheme 43) [69,70]. It should be noted that the yields of reaction strongly depend on the applied bromination method of pyrene, and the authors did not report the ratio of the starting material mixture.

**Scheme 44:**
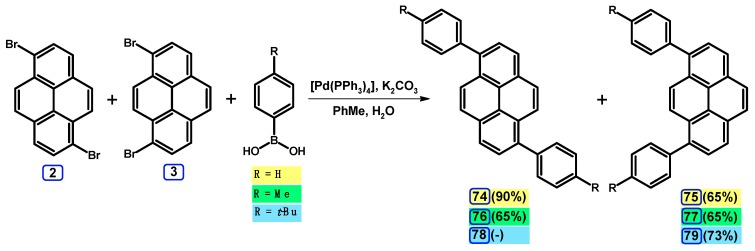
Suzuki-Miyaura coupling reaction with a mixture of dibromo isomers [6,9].

Zhonghai Ni et al. used a mixture of 1,8-dibromopyrene (85%) and 1,6-dibromopyrene (15%) in the Suzuki-Miyaura coupling reactions with phenylboronic acid (for **74** and **75**) or 4-substituted-phenylboronic acids (for **76**–**79**). Target compounds were isolated by crystallization or column chromatography with yields in the range of 65–90%, expressed per the starting material (Scheme 44) [6,9].

**Scheme 45:**
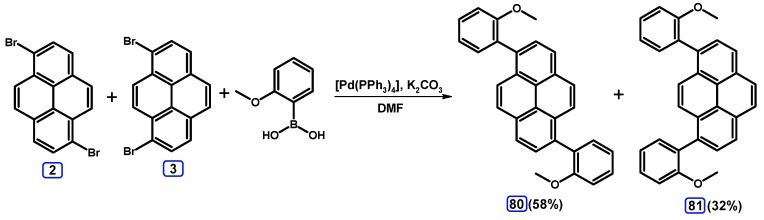
Suzuki-Miyaura coupling reaction resulted in **80** and **81** [71].

Lawrence T. Scott et al. also applied a mixture of 1,6- and 1,8-dibromopyrene (the ratio of the starting material mixture is unknown) in the Suzuki-Miyaura coupling reaction with 2-methoxyphenylboronic acid, and the obtained isomers **80** and **81** were separated by a simple treatment with acetone, resulting in the products with 58% (**80**) and 32% (**81**) yields (Scheme 45) [71].

**Scheme 46:**
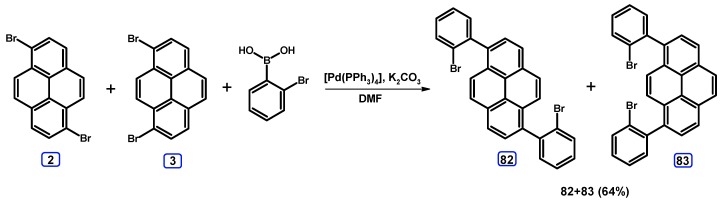
Suzuki-Miyaura coupling reaction of a mixture of dibromopyrenes with 2-bromophenylboronic acid [71].

They also reported another example where a mixture of the two dibromopyrenes reacted with 2-bromophenylboronic acid at conditions, which resulted in a mixture of diindenopyrenes, but there was no possibility to separate the isomers. Therefore, a two-step variant was applied, obtaining pure isomers **82** and **83** with a total reaction efficiency of 64% (Scheme 46), which were reacted further in the direction of diindenopyrenes [71].

## 3. Acetylpyrenes

**Scheme 47:**
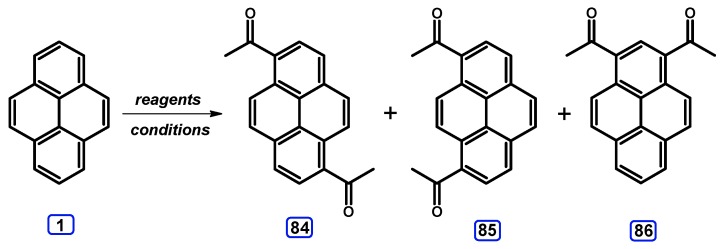
Acylation reaction of pyrene.

Apart from 1,6- and 1,8-dibromopyrenes, the significant role as a starting material in the synthesis of 1,6-, 1,8-, and 1,3-disubstituted pyrenes by heteroaryl groups play acetylpyrenes due to the wide possibility of functionalization of an acetyl group [72]. Their synthesis is based on the acylation of pyrene using acetyl chloride (AcCl), what resulted in disubstituted and various isomers of acetylpyrenes (Scheme 47).

Reaction conditions reported in the literature are based on AcCl with AlCl_3_ as a catalyst in carbon disulfide, which results in 1,8-diacetylpyrene **85** with the highest yields up to 46%, followed by 1,6-isomer **84** and 1,3-diacetylpyrene **86**. [73,74,75] Separation of the isomers can be achieved by crystallization or column chromatography (Table 3). Moreover, application of the ionic liquid (1-methyl-3-ethylimidazolium chloride) in the acylation of pyrene was described by Martyn J. Earle et al., which resulted in a mixture of 1,6- and 1,8- isomer with total reaction efficiency of 55% [76].

**Scheme 48:**
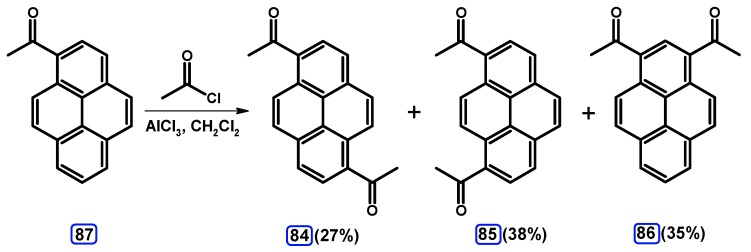
Acylation reaction of 1-acetylpyrene **87** [36].

Masahiro Minabe and co-workers reported the way of synthesis of diacetylpyrenes starting from 1-acetylpyrene **87**, which resulted in isomers **84** (27%), **85** (38%), and **86** (35%) (Scheme 48) [36].

### Condensation Reactions with Acetylpyrenes

**Scheme 49:**
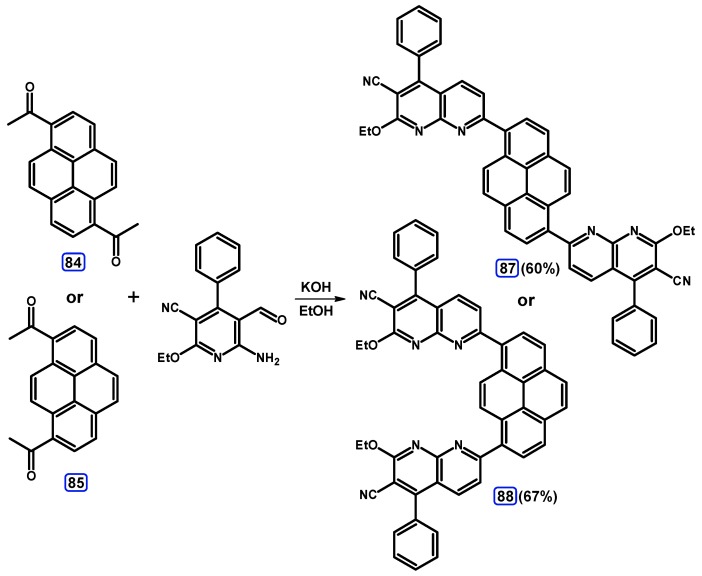
Friedländer condensation resulted in compounds **87** and **88** [77].

Pure 1,6- and 1,8-diacetylpyrene (**84** and **85**) were used by Carlos Peinador and co-workers in the Friedländer condensation reaction with 2-amino-5-cyano-6-ethoxy-4-phenylpyridine-3-carbaldehyde, which resulted in 1,6- and 1,8-di(1,8-naphthyridyn-20-yl)pyrenes with yields of 60% for **87** and 67% for **88** (Scheme 49) [77].

**Scheme 50:**
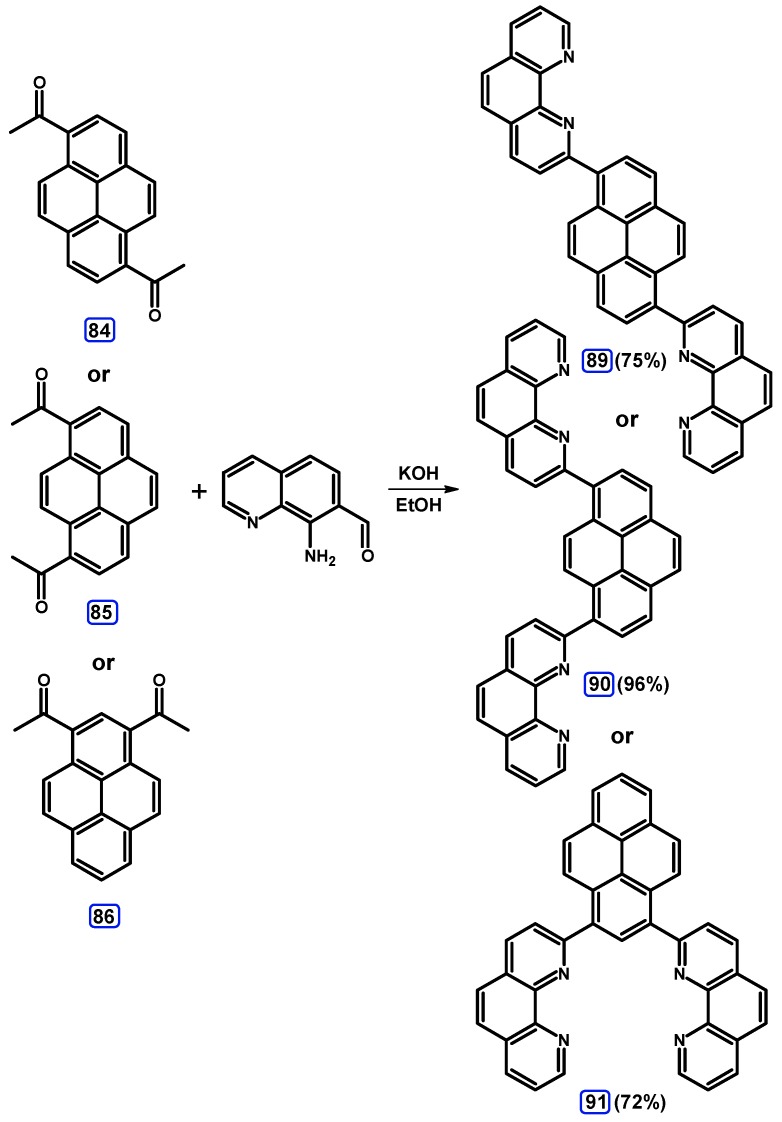
Friedländer reaction resulting in pyrenes with phenanthrolinyl units [74].

As the result of the Friedländer reaction between 1,3-, 1,6- and 1,8-diacetylpyrene **84**–**86** with 8-amino-7-quinolinecarbaldehyde, Randolph P. Thummel et al. obtained bis(2′-[1′,10′]phenanthrolinyl)pyrenes **89**–**91** with high yields up to 96% (Scheme 50) [74]. They were applied as ligands in the synthesis of dinuclear ruthenium complexes.

**Scheme 51:**
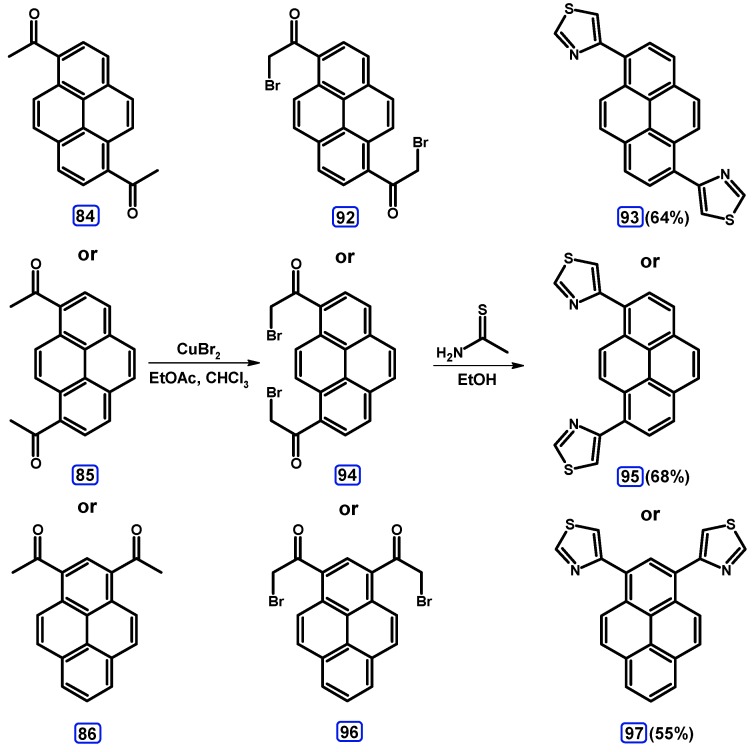
Hantzsch condensation resulting in molecules **93**, **95**, and **97** [78].

In 2016, Mahesh Hariharan et al. described the way of synthesis of bisthiazolylpyrenes starting from the pure isomers of acetylpyrenes **84**–**86**, which were then reacted with copper(II) bromide resulted in bromoacetylpyrene derivatives **92**, **94**, and **96**. Intermediates were used in the Hantzsch condensation reaction between thioacetamide and appropriate bis(bromoacetyl)pyrene, which obtained target molecules **93**, **95**, and **97** with 64%, 68%, and 55% yields, respectively (Scheme 51) [78]. It should be noted that, as the result of all presented condensations reactions, isomers with substitution pattern 1,8 were obtained with the highest yields.

## 4. 1,3-Disubstituted Pyrene

**Scheme 52:**
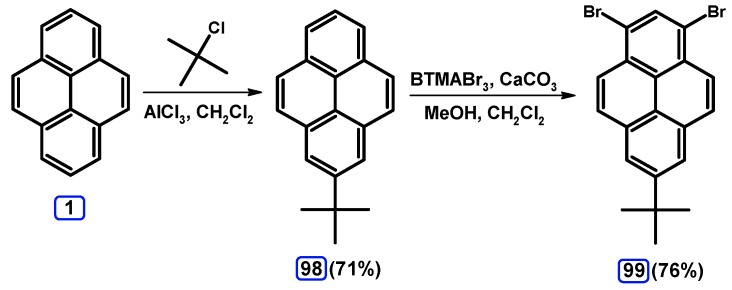
Introduction of protecting *tert*-butyl group [79].

The most challenging of disubstituted pyrenes are the derivatives with the 1,3-substitution pattern, as presented earlier. Apart from their synthesis starting from 1,3-diacetylpyrene, which allows for the introduction of a limited group of substituents into the pyrene structure at positions 1 and 3, another approach is presented in the literature. Takehiko Yamato et al. reported 1,3-diphenylpyrene **101**, which was obtained in a multistep procedure [79]. As the first step, the introduction of the protecting group was achieved by the alkylation of pyrene at the 2-position by *tert*-butyl chloride, resulting in molecule **98** with a 71% yield [79]. The intermediate **98** was brominated by benzyltrimethylammonium tribromide (BTMABr_3_), which led to the synthesis of 1,3-dibromo-7-*tert*-butylpyrene **99** with a 76% yield (Scheme 52).

**Scheme 53:**
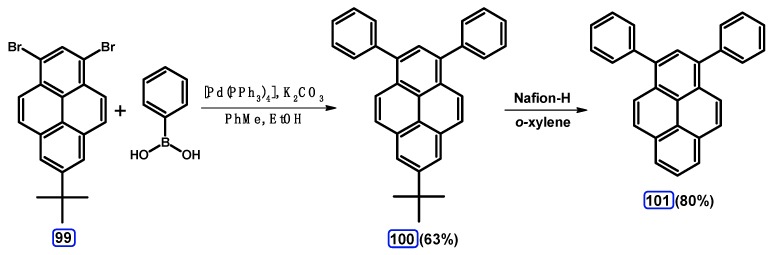
Synthesis of 1,3-diphenylpyrene **101** [80].

Compound **99** was used in the Suzuki-Miyaura coupling reaction with phenylboronic acid and molecule **100** containing phenyl groups at positions 1 and 3, and a protecting group at 7-position was obtained. Removing the protecting *tert*-butyl was conducted by using Nafion-H as a catalyst, which resulted in compound **101** with an 80% yield (Scheme 53) [80].

## 5. Synthesis of 1,3,6,8-tetrasubstituted Starting from Disubstituted Pyrenes

**Scheme 54:**
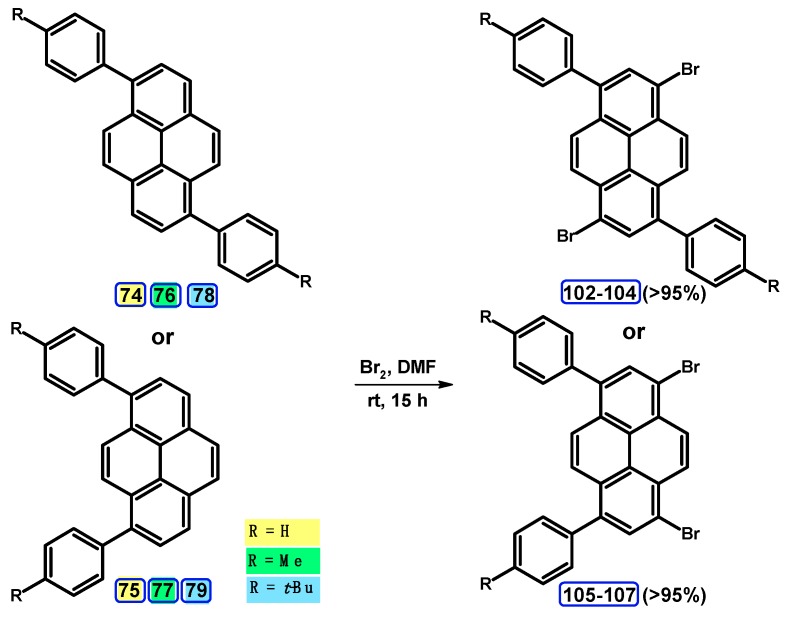
Bromination of disubstituted pyrenes **74**–**79** by Br_2_/DMF [6,9].

**Scheme 55:**
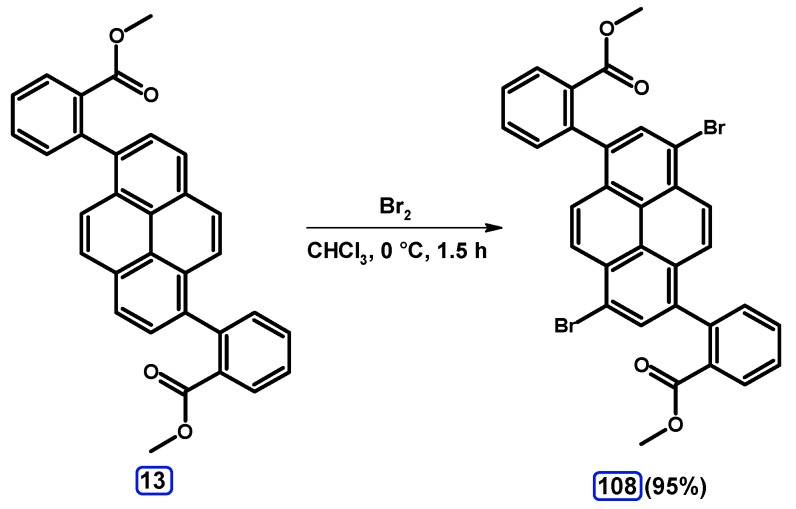
Bromination of disubstituted pyrene **13** by Br_2_/CHCl_3_ [44].

In many cases, disubstituted pyrenes by (hetero)aryl groups act as substrates in the subsequent reactions: functionalization of already introduced substituents or the introduction of other groups into the pyrene structure at unoccupied positions, especially at the non-K region, which is possible by the introduction of bromine atoms. Brominating agent bromine solution in DMF or CHCl_3_ was used, which resulted in products with yields above 95% (Scheme 54 and Scheme 55) [6,9,44].

**Scheme 56:**
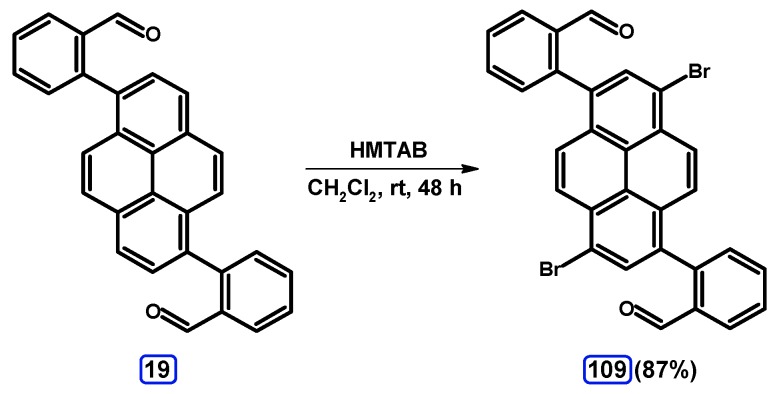
Bromination of disubstituted pyrene **19** by HMTAB/CH_2_Cl_2_ [13].

**Scheme 57:**
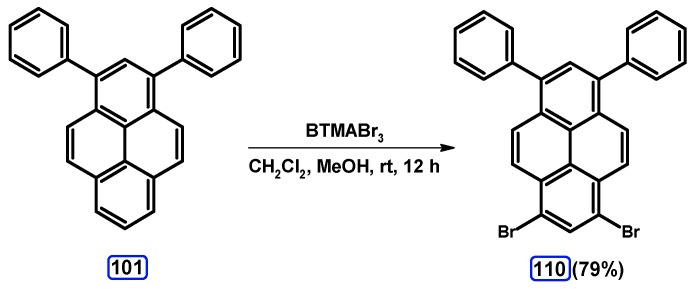
Bromination of disubstituted pyrene **101** by BTMABr_3_/CH_2_Cl_2_ + MeOH [80].

What is more, another approach to bromination of disubstituted pyrene was achieved using the hexamethylenetetramine-bromine complex (HMTAB) (Scheme 56) [13] and benzyltrimethylammonium tribromide (BTMABr_3_) (Scheme 57) [80].

**Scheme 58:**
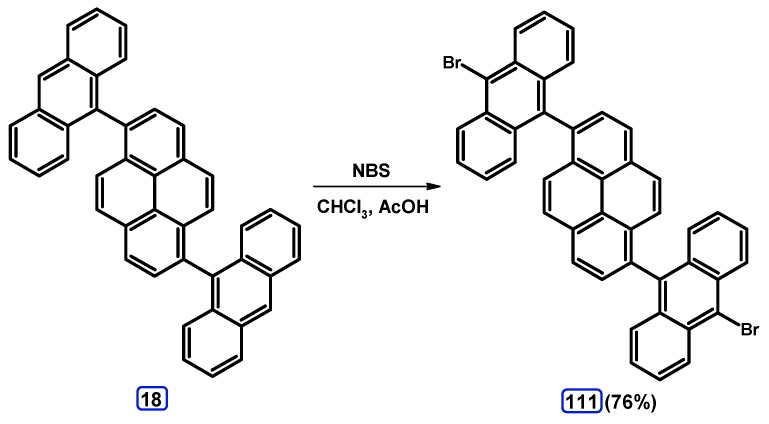
Bromination of disubstituted pyrene **18** by NBS/CHCl_3_+AcOH [7].

Unlike bromination using *N*-bromosuccinimide (NBS), the unoccupied positions of disubstituted pyrene remained unchanged, which was reported for 1,6-di(anthracen-9-yl)pyrene **18** (Scheme 58) [7].

## 6. Summary

The review of structures of 1,3-, 1,6-, and 1,8-disubstituted pyrenes by (hetero)aryl groups and the methods for their synthesis revealed that the number of 1,6-isomer derivatives is the highest and compounds are preferably obtained using the Suzuki-Miyaura coupling reaction. The main reason for taking interesting in those compounds is connected with their optical and photophysical properties, which make them potential materials for broadly defined organic electronics. The wide possibility of obtaining of 1,6- and 1,8-dibromopyrene, unlike 1,3-dibromopyrene, showed that, in the case of 1,3-isomer, indirect methods must be applied. Moreover, acylation of pyrene allows 1,3-, 1,6-, and 1,8-isomers to be obtained, which can be successfully used in condensation reactions that result in products with high yields. I believe that, as the results of the presented systematization and described diversity in the area of disubstituted pyrenes at the non-K region, the expected direction in pyrene chemistry will be followed.

## Figures and Tables

**Table 1:** The reported bromination reactions of pyrene.

Entry	Brominating Agent	Solvent	Reaction Conditions	Yield [%]	
				1,6-	1,8-
1[19]	Br_2_	CH_2_Cl_2_	rt, 24 h	15	-
2[20]	Br_2_	CH_2_Cl_2_	rt, 2 h	50	-
4[21]	Br_2_	CH_2_Cl_2_	rt, 20 h	25	9
4[22,23]	Br_2_	CHCl_3_	rt,17 h	33	-
5 [13]	Br_2_	CHCl_3_	rt, 24 h	36	-
6[24]	Br_2_	CHCl_3_	rt, 5 h	14	-
7[25]	Br_2_	CHCl_3_	rt, 17 h	14	6
8[26]	Br_2_	CCl_4_	110 °C, 12 h, darkness	63	-
9[27]	Br_2_	CCl_4_	rt, 16 h	21	-
10[18]	Br_2_	CCl_4_	rt, 17 h	44	45
11[28]	Br_2_	CCl_4_	rt, 17 h	61	-
12[29]	Br_2_	CCl_4_	rt, 24 h	28	13
13[30]	Br_2_	CCl_4_	rt, 48 h	38	-
14[31]	Br_2_	CCl_4_	rt, 54 h	25	50
15[6,9]	Br_2_	CS_2_	rt, 17 h	15	85
16[32]	DBMH	CH_2_Cl_2_	rt, 1 h	97	
17[33]	BTMABr_3_ + ZnCl_2_	CH_2_Cl_2_, MeOH	rt, 16 h	quant.	
DBMH–1,3-dibromo-5,5-dimethylhydantoin BTMABr_3_–benzyltrimethylammonium tribromide					

**Table 2:** Reported bromination reactions of 1-bromopyrene.

Entry	Brominating Agent	Solvent	Reaction Conditions	Yield [%]	
				1,6-	1,8-
1[35]	KBr + NaClO	HCl, MeOH	rt, 24 h	43	
2[36]	Br_2_	CH_2_Cl_2_	rt, 6 h	35	36

**Table 3:** Reported conditions of acylation reaction.

Entry	Reaction Conditions	Yield [%]		
		1,6-	1,8-	1,3-
1[73]	AcCl, AlCl_3_, CS_2_, rt, 3 h	9.6	37.5	9.4
2[74]	AcCl, AlCl_3_, CS_2_, rt, 2 h	25.0	46.0	11.0
3[75]	AcCl, AlCl_3_, CS_2_, rt, 2 h	14.8	40.2	12.3
4[76]	AcCl, [emim]Cl–AlCl_3_, rt, 2 h	55.0		-
[emim]Cl–1-methyl-3-ethylimidazolium chloride				
